# Asymptomatic Visceral Leishmaniasis, Northern Israel

**DOI:** 10.3201/eid0903.020297

**Published:** 2003-03

**Authors:** Irit Adini, Moshe Ephros, Jacopo Chen, Charles L. Jaffe

**Affiliations:** * The Hebrew University–Hadassah Medical School, Jerusalem, Israel; † Technion-Israel Institute of Technology, Haifa, Israel

**Keywords:** Visceral leishmaniasis, *Leishmania infantum*, ELISA, antibodies, epidemiology, dispatch

## Abstract

Asymptomatic human visceral leishmaniasis was identified in Israel by using an enzyme-linked immunosorbent assay. Positive serum samples were more prevalent in visceral leishmaniasis–endemic (2.97%) compared to nonendemic (1.01%) regions (p=0.021). Parasite exposure was higher than expected, despite the small number of clinical cases, suggesting factors other than infection per se influence clinical outcome.

Human visceral leishmaniasis (HVL), caused by the *Leishmania donovani* complex, is lethal if not promptly diagnosed and treated (*1*). Yet in the Mediterranean and Middle East, only a small percentage of infections progress to clinical disease (*2*,*3*). HVL in this region is primarily a disease of young children; however, its epidemiology is changing. In southern Europe, 50% of new cases are in adults coinfected with HIV (*4*).

HVL is endemic in northern Israel; 68 cases, mostly from Arab villages, were documented from 1960 to 1989 **(***5*). Since 1993, a drastic reduction in the disease was observed, despite the identification of nine active sites of canine visceral leishmaniasis (VL) in this region (G. Baneth and C. L. Jaffe, unpub. data). Recent studies have identified an emerging focus of human and canine disease near major population centers in central Israel and the Palestinian Authority (*6*,*7*). In this study, HVL seroprevalence in northern Israel was compared with that in a region free of clinical disease.

## The Study

Prevalence of asymptomatic disease was examined by random cluster sampling of serum samples from 12 sites (494 serum samples) in a region presumed to be non–HVL-endemic (i.e., HVL has not been reported) and 11 sites (2,086 serum samples) in the HVL-endemic northern region, where the disease was previously reported in 8 of 11 sites (Figure). Coded samples were analyzed blindly by enzyme-linked immunosorbent assay on *L. donovani* antigen for anti-leishmanial antibodies (*5*). Each plate was read when the positive control serum (1/1,000 dilution) absorbance **_λ_**_405 nm_ = 1.0–1.2. Most serum samples were from women ages 18–45. The mean ages for patients in the non–disease-endemic and HVL-endemic regions were 36 and 30 years, respectively (range <1 to >75). No symptomatic HVL was diagnosed during the study.

**Figure:**
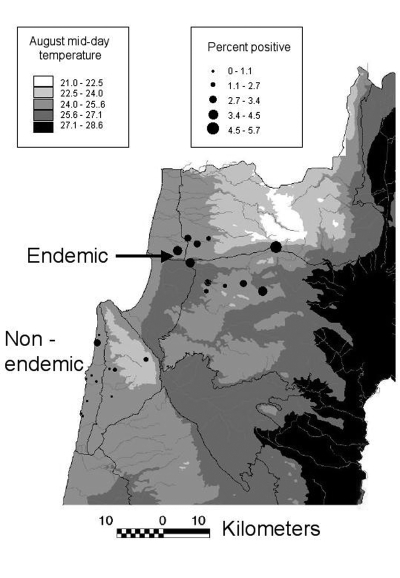
Percentage of positive serum samples and average mid-day temperatures, visceral leishmaniasis study sites, northern Israel

The mean absorbance for serum samples from HVL-endemic areas (0.0956; 95% confidence interval [CI] 0.0926 to 0.0986) was significantly higher (p=0.0001; unpaired t test with Welch’s correction) than that for serum samples from non–disease-endemic areas (0.0832; 95% CI 0.0779 to 0.0885). The percentage of positive samples (mean of the non–HVL-endemic population + 3 standard deviations) was significantly higher (p=0.021, chi-square test) for the endemic, 2.97% (n=62) than for the non–HVL-endemic region, 1.01% (n=5). Age was directly standardized by examining the percent positive serum samples in children ≤14 years of age, who are most likely to have clinical HVL, and study participants (>14 years). The standardized prevalence ratio of positive serum samples in the HVL-endemic to non–HVL-endemic regions was 2.90. Western blotting was used to confirm asymptomatic VL (*8*). Strong reactions were observed to 14- and/or 18-kDa antigens with all positive samples; weak or no bands were observed with negative samples.

The percentage of positive serum samples for each site is shown (Figure). In the non–HVL-endemic region, most serum samples collected (64.6%) came from Atlit (Jewish, n=146) and Isfiya (Arab, n=173). The remaining serum samples (n=175) originated from 10 sites, with a maximum of 39 samples per site. Only 1.1% of these samples (2/175) were positive, a value similar to that found for the larger towns of Atlit and Isfiya (p=1.0, Fisher exact test). Essentially no difference in the percentage of positive serum samples (p=1.0, Fisher exact test) was found between non–leishmaniasis-endemic Arab (Isfiya, 1.2%) and Jewish (Atlit, 0.7%) towns, even though residents of Isfiya are more likely to visit villages where the disease occurs.

Large numbers of samples (>140/site) were collected from 9 of 11 HLV-endemic sites. The percentage of positive samples from Arab areas (3.1%) was not significantly higher (p=0.46, chi-square test) than for samples from Jewish areas (1.8%), even though most HVL case-patients in this region are from Arab villages. However, the percentage of positive serum specimens in 7 of 11 HVL-endemic sites was three- to six-fold the average percentage found in the non–HVL-endemic region. The number and percentage of positive HVL-endemic serum samples during the second year were almost twice (3.9%; p=0.038, unpaired t test) the figures observed for the first year (2.2%). The opposite trend was found in the non–HVL-endemic region, where the number and percentage of positive samples decreased from 1.9% to 0.3% (p=0.044) in the first and second year, respectively. Nine of the 11 HLV-endemic sites showed increases in the percentage of positive serum samples during the second year.

During the 1960s, 45 cases of VL were diagnosed in western Galilee; more than half were from eight sites, all Arab villages, included in this study (*5*). Since then, the incidence of HVL in northern Israel has decreased: only three cases have been diagnosed in the last 7 years. This finding is not due to the absence of parasite transmission, since canine VL remains common (G. Baneth and C. L. Jaffe, unpub. data). While HVL is decreasing in northern Israel, the disease has emerged in central Israel, where numerous dogs and seven persons have been diagnosed with VL since the index HVL case in 1994 (*6*). Villages in both regions are rural, and stray dogs and jackals are frequently observed. The conditions contributing to the changes in disease epidemiology in Israel are unknown but are likely multifactorial. Studies in Brazil on risk factors associated with asymptomatic infection concluded that infection is associated with age (≥2 years), location of dwellings, and presence of relatives with acute VL. Malnutrition was not associated with infection (*9*). A higher standard of living and health was also postulated to be involved in decreased VL incidence over the past 10 years (*10*). During the last decade, improvements in the standard of living, health care, and infrastructure of Arab villages in Israel have taken place. Though data are not available for most study sites, the percentage of seropositive persons in Yirka, for example, decreased from 10% in 1989 (*5*) to 1.5% in the current study (1994–1996), suggesting that exposure to parasites in Yirka has decreased during the last decade.

The percentage of seropositive people is significantly greater in the HVL-endemic northern than the non–HVL-endemic coastal and Carmel Mountain regions. The presence of positive samples in the non-HVL–endemic region suggests that parasite transmission occurs, even though HVL has not been reported. Since people from this region frequently travel to northern Israel, we cannot exclude the possibility that they were infected there. Although the incidence of the disease is lower in Israel than in other HVL-endemic regions, our results suggest that cumulative exposure to the parasite and asymptomatic infection in Israel are more frequent than otherwise predicted, based on the yearly incidence of VL. These data will be important in understanding the spread of human and canine disease into new regions, transmission by blood transfusion, and the potential risk for VL/HIV coinfection.
